# General Index

**Published:** 1790

**Authors:** 


### GENERAL INDEX

TO THE

### LONDON MEDICAL JOURNAL.

[The Roman Numbers refer to the Volume; the Arabic the Page.]

### A.

ABDOMEN, cafes of large tumours found in the cavity of, VII. 287, 298
————, abfcefs of, —— — I. 328. XI. 138
Abfcefs, lumbar, cafe of, —— —— VII. 1[illegible]
——— of the liver, ———, —— —— ibid. 22
——— of the belly, communicating with the inteftine,———, XI. 138
——— of the calf of the leg, which terminated fatally,———, I. 330
Abfceffes, in different parts, after a fore throat, cafe of, ibid.
Achillis, tendo, divifion of, ——— II. 19. VIII. 304
Acid, vitriolic, in a native concrete ftate, defcribed, — II. 44
Acrell, Olaus, furgical obfervations by, II. 208. III. 1, 144
Acta, Havnienfia, abftract of, ——— [illegible]. 377
——, Petropolitana, ———, ——— II. 26. IV. 129
Adanfon, M. on the gum trees of Senegal, —— IV. 28
Aery, Dr. Tho. his remarks on the hydrocephalus internus, I. 424
Æther, vitriolic, obfervations relative to, —— VI. 53, 337
Aikin, Dr. J. his edition of Dr. Lewis's Materia Medica, ibid. 89
Air, obfervations on, —— —— I. 51. VI. 171
——, emitted by perfpiration, account of, —— II. 183
——, fixed, remarks on, —— I. 56, 440. IV. 74, 238
——, inflammable, obfervations relative to, — II. 424. IV. 6
——, purification of, by vegetables, ——— II. 1
Aitken, John, outlines of the theory and cure of fever, I. 366
Alanfon, Edward, on amputation, —— — IV. 153
Alcock, Dr. his theory of epidemics, ——— II. 382
Aleppo, account of a fingular difeafe at, — IV. 366
Algiers, obf. on the modes of inoculating there, — XI. 141
Alkali, volatile, its effects in gangrene, —— ibid. 169
————————— as a ftyptic, —— ibid. 315
Aloes, preparation of, in Jamaica and Barbadoes, VIII. 219, 422
Alum,

II.

Alum, discourse on the history of, —— III. 256

——, large doses of, taken with impunity, —— II. 86

——, roch, etymology of, ——

Amar, Don Joseph, on small pox, I. 72; on eruptive diseases, *ib.* 142; on diseases of the breast, *ib.* 143

Amaurosis, observations relative to, —— II. 10. IX. 389

Amenorrhœa, facts and obs. relative to, —— I. 92. XI. 230

Amputation, remarks on, IV. 153, 273, 406. VII. 225, 377. VIII. 142. IX. 54, 117, 223

X. 371

Ancients, their obscure accounts of some diseases, —— *ibid.* 424

Anderson, James, his account of a monster, ——

——, Dr. J. on the Tanjore antidotes for venomous bites, *ibid.* 285, 289

XI. 182

——, Thomas, pathological obs. on the brain, —— I. 418

Andree, John, account of an elastic trochar, —— *ibid.* 433

Andry, M. remedy employed by him in gonorrhœa, —— III. 288

——, on the hydrophobia, —— ——

Aneurism, obs. relative to, I. 334. II. 116. III. 17. VI. 213. VII. 104, 391, 401. VIII. 126. IX. 142

XI. 190

Angina, maxillaris, account of, —— —— III. 308. V. 162

——, pectoris, obs. relative to, —— —— I. 217

——, trachealis, treatise on, ——

Angustura bark, accounts of, —— X. 154, 158. XI. 38, 331

Animal magnetism. See Magnetism.

IV. 48

Anthrax, observations on a species of, ——

Anthelmintics, obs. relative to, I. 95. II. 175. III. 314. VIII. 253, 254, 256, 285

II. 40

Antimony, wine of, mode of preparing it, —— I. 12

Antispasmodics, remarks on, —— —— III. 26

Anus, case in which it was deficient, —— *ibid.* 356

Arm, fractured, remarkable operation on, —— X. 236

Arnica montana, its medicinal effects, —— VIII. 229

Arnotta, account of, —— —— III. 146

Arsenic recommended in the cure of cancers, ——

—————— intermittents, VII. 192. VIII. 191. IX. 48

VIII. 385

Artery, femoral, case of a dilatation of, —— XI. 357

——, ulnar, case of a wound of, —— I. 23

Assafœtida, its use in carious ulcers, —— VII.

——, a plant that yields it described, ——

Ascites. See Dropsy.

IV. [illegible]

Asphaltites, lake, analysis of its water, —— I. 397

Asphaltum, oil of, recommended in phthisis, —— *ibid.* 370

Asti, Felix, on the poison of mad animals, —— IX. 405

Astragalus Exscapus, its effects in lues venerea, —— XI. 317

——, botanical description of, ——

### B.

X. 178

Baillie, Dr. M. case of transposed viscera, —— *ibid.* 322

——, on a change of structure in the ovarium,

Baldaffari, Jof. of a native, concrete vitriolic acid, — III. 44
Bandages, elaftic, remarks on, ———— IX. 44
Banks, Sir Jof. Reliquiæ Houftounianæ, —— V. 253
————, defcription of a new fpecies of cinchona, VI. 177
————, remarks on a plant that yields affafœtida, VII. 71
Bark, Peruvian, condemned, —— —— III. 182
————, red, accounts of, II. 340. III. 248, 313, 432
————, natural and chemical hiftory of, IV. 304, 428
————, its folubility fuppofed to be increafed by magnefia, VII. 254, 419
————, effential falt of, what fo called, — ibid. 262
————, a new extract of, defcribed, —— XI. 67
——, accounts of a new fpecies of, X. 154, 158. XI. 38, 331
Bartholin, Tho. his defcription of a monfter, — X. 425
Baths, Ruffian, account of, —— —— V. 355
——, warm, ufe of, in allaying morbid irritability, — I. 182
Bayley, Richard, on angina trachealis, —— ibid. 217
Beard, growth of, in a female, —— — IV. 96
Beau, J. F. Le, anecdotes of, —— — III. 151
Beccaria, Father, fome account of, —— — V. 111
Beckmann, John, on the hiftory of alum, —— II. 85
Belladonna, its effects in cancers, II. 16; in hydrophobia, III. 199
————, berries of, their effects, —— VI. 425
Bellofte, M. his recommendation of walnut leaves in ulcers, X. 321
Benjamin tree, botanical defcription of, —— IX. 80
Berger, Mr. on faltpetre, —— —— III. 22
Bergius, P. J. on the ledum buxifolium, —— II. 38
———— effects of foap and lime water in cafes of calculus, —— —— III. 24
Bergman, Thorb. opufc. phyf. et chem. I. 51, 73. III. 331. IV. 439
————, de diverfa quant. phlogifti in metallis, II. 141
————, letter from, —— III. 89
Bernouilli, Daniel, anecdotes of, —— IV. 426
Beunie, M. de, on the poifonous effects of mufcles, III. 134
Bew, Dr. Geo. cafe of a perfon who fwallowed a fhirt pin, IV. 77
————, experiments on latent heat, —— V. 205
————, of an epidemic catarrh, —— IX. 354
Bewley, Mr. his mephitic julep, how prepared, — II. 140
Bezoar, account of, —— —— III. 143
Binney, Barn. cafe of a gun-fhot wound, —— VII. 294
Birds, receptacles of air in, injected, —— III. 198
Births, numerous, obfervations relative to, —— X. 83
Bifmuth, magiftery of, its effects defcribed, VI. 406 VII. 321
Blackburne, Dr. Tho. cafe of hydatids voided with the urine, I. 125
———— uterine hæmorrhage, II. 122
————, anecdotes of, —— III. 321
————, Dr. W. account of a difeafed uterus, — VIII. 60
————, effects of a large dofe of emetic tartar, IX. 61
Blackhall, And. account of, —— — I. 282
———— preparations in his collection, ibid. 359
Bladder, urinary, effects of mercury in a fpafmodic affection of, X. 356

Bladder,

Bladder, urinary, caſes in which it was punctured above the pubis, XI. 7, 15, 109

——, ſingular affection of, —— ibid. 121

——, obſ. on the puncture of, through the rectum, ibid. 349

Blagden, Dr. Charles, on the congelation of quickſilver, V. 233

Bland, Dr. Robert, inquiries relative to population, —— I. 355

——, inſtance of a fœtus affected with ſmall pox, II. 204

——, remarks on convulſions during parturition, ib. 328

——, midwifery tables, —— III. 113

——, caſes of hæmaturia, —— IV. 282

Blane, Dr. Gilbert, account of a hurricane, —— I. 132

——, on the health of ſeamen, —— II. 61

Bleeding, topical, its efficacy in wounds of the head, —— ibid. 113

——, abuſe of, in forced marches, —— ibid. 110

——, miſtakes of the ancients relative to, —— III. 264

——, remarks on the ſubject of, —— X. 139

Blind perſons, intereſting accounts of, —— III. 21

Blizard, William, on the fiſtula lachrymalis, —— I. 62

—— external uſe of emetic tartar, VIII. 57

—— uſe of electricity in deafneſs, XI. 31

+> Bloch, M. on inteſtinal worms, —— VI. 76

Blunt, Robert, of a painful affection of the face, —— VII. 115

Bones, their ſenſibility in a diſeaſed ſtate, —— III. 223

——, fragility of, —— —— IV. 309

——, ſpecifically lighter than water, inſtance of, —— ibid.

——, ſoftneſs of, caſe of, —— VI. 288. VIII. 67, 70

Bonomo, Sig. his account of the itch inſect, —— IX. 31

Bordenave, M. on the Cæſarean ſection, —— II. 182

—— motion of the ribs, —— IV. 32

Bouſſelin, M. on necroſis, —— —— VII. 263

Boutan, account of the diſeaſes of, —— XI. 274

Boys, William, caſe of a child who ſwallowed a pin, VI. 400

Brain, concuſſion and ſuppuration of, —— I. 324

——, wound of, by a muſket ball, —— ibid. 325

——, diſeaſes of, —— III. 4, 146. XI. 182

——, appearances of, in maniacal ſubjects, —— VI. 159

Brandé, Auguſtus, on the Anguſtura bark, —— XI. 38

Brandis, Joſeph, caſe of a gun-ſhot wound, —— VII. 135

—— mortification of the leg, - VIII. 123

—— exfoliation of the jaw bone, ibid. 296

Bread, freſh baked, bad effects of, —— I. 333

Brereton, Owen Saluſbury, of the effects of lightning, II. 192

Briſtol water, account of, —— —— ibid. 324

Brodbelt, Dr. Francis Rigby, caſes by, —— VI. 367, 371, 372

Broughton, Dr. Arthur, on the catarrh of 1782, —— III. 297

Brouſſonet, P. M. A. Ichthyologia, —— ibid. 326

Brumwell, Mr. on the effects of belladonna, —— VI. 425

Bruſſells, Memoirs of the Imperial Academy at, —— III. 133

Buboes, venereal, how to be treated, —— II. 242

Burning glaſs, its uſe in ſurgery, —— III. 274

Butini,

+> Blood in a living Animal contains no air in an expansible state, Expts to prove this repeated by Dr. Simmons & Mr. Cline. IV. p. 272.

Butini, Peter, on magnesia, — — II. 358

—— on gastric juice and magistery of bismuth, VI. 404

Buxton, accounts of, — — I. 276, 434

Byssus, new species of, described, — III. 430. IV. 421

### C.

Cæsarean operation, cases of, IV. 94, 239, 378. VI. 372 XI. 146

Cadet, M. on the acids of phosphorus and sugar, — II. 186

Calamus aromaticus, its efficacy in intermittents, — I. 388

Calculus, urinary, obs. relative to, II. 16, 24. V. 387. XI. 237

—— in the pulmonary artery, instance of, — X. 347

—— extracted from a cyst in the neck, — XI. 362

Calomel, modes of preparing it, — II. 52. III. 283

——, good effects of, in a case of obstruction of the menses, VII. 413

Cammel, Mr. case of extra-uterine fœtus, — V. 396

Camper, Peter, his account of the rhinoceros, — II. 30

—— a disorder of horned cattle, III. 356. IV. 386

—— red bark, — III. 432

—— essay on the best form of shoes, — IV. 343

—— obs. on lithotomy, — X. 162

Camphor, its effects in a case of mania, — VI. 120

——, remarkable effects of, on a dog, — *ib*. 426

Cancers, ointment for, — — I. 193

——, cases and obs. relative to, I. 206. II. 364. III. 4, 5, 6, 146. IV. 406. V. 109. X. 40, 53

Cancerous breast, improved mode of amputating, — IV. 406

Cantharides, tincture of, mode of preparing it, — II. 39

——, substitutes for, described, — V. 130, 131

Cantharus, a Swedish measure, described, — I. 75

Caoutchouc, its generical character, — VI. 315

Capsicum, its properties described, — VIII. 235

Carex arenaria, substituted for sarsaparilla, — VI. 411

Caries of the spine, case of, — — I. 271

—— scull, — — I. 334. II. 112

Carlisle, account of the jail fever at, — III. 268

Caruncles of the urethra, remarks on, — II. 244

Cashew tree, described, — — *ibid*. 222

Cascarilla, observations relative to it, — VIII. 250

Cassavi root, its use in ulcers, — — VI. 393

Castor oil, observations relative to, — VIII. 278

Castration, for the cure of hernia, condemned, — III. 273

Catalepsy, cases of, — — *ibid*. 144, 376

Catamenia. See Mensfes.

Cataract, obs. relative to, — III. 7. IX. 109. X. 395

Catarrh, epidemic, obs. relative to, III. 152, 202, 294, 297, 318. IV. 434, 435, 438. IX. 335, 342, 354

Catgut, its use in obstructions of the urethra, — IX. 378

Cavallo, Tiberius, on medical electricity, — I. 246

Cavear, how prepared, — — *ib*. 26

Cavendish,

Caveudiſh, Henry, on the freezing of quickſilver, — V. 23[illegible]

———, experiments on air, — VI. 171

Cauſer, John, caſe of a fracture of the ſcull, — VII. 152

Cautery, actual, origin of, — — III. 262

———, applied to the head, fatal effects of, - IV. 353

Cawley, Dr. Thomas, account of different caſes, — VI. 366

———, obſ. on dyſentery, — VII. 337

——— diabetes, — IX. 286

Cevadilla, natural hiſtory of, — — V. 135

Chandler, Dr. Benj caſe of a ſtone of the bladder, ibid. 387

Chavaſſe, Nich. on the cure of dyſentery, — ibid. 297

——— medical and ſurgical uſe of cold water, VII. 123

——— a remarkable diſeaſe of the heart, — ibid. 407

Chaulnes, M. le Duc de, on the fuſible ſalt of urine, V. 225

Cherry ſtone extracted from an abſceſs of the abdomen, II. 337

Cheſham, epidemic ſore throat at, deſcribed, — X. 7

Cheſsher, R. caſe of luxation of the os humeri, — VIII. 189

Cheſter, Reports of the Small-Pox Society at, II. 59. III. 427

Cheſton, R. B. on polypoſe concretions of the heart, VI. 213, 225

Children, management of, obſervations on, — I. 171

———, diſeaſes of, ibid. 172, 408. III. 289. VI. 318. XI. 374

Chineſe, their mode of cultivating muſhrooms, — II. 30

——— uſe of different agarics as food, — ib. 31

Chocolate tree, deſcription of it, — VIII. 288

Chordee, remarks on, — — ibid. 242

Chorea ſancti viti, caſe of, — — VII. 187

Chriſtie, Thomas, obſervations on pemphigus, — X. 361

Chryſtalline humour, method of extracting it, — ibid. 416

Cicuta, remarks on its medicinal effects, — II. 293. V. 86

Cinchona, different ſpecies of, deſcribed, VI. 175. VIII. 39, 240

Cinnamon tree, cultivated in Jamaica, — VIII. 266

Clark, Thomas, caſe of epilepſy, — I. 428

Clarke, John, caſes of laborious parturition, — VII. 40

———, on the compreſſion of the umbilical cord, VIII. 182

———, Dr. Joſ. on the regiſtry of the Lying-in Hoſpital at Dublin, IX. 179

——— properties of human milk, XI. 71

Clauſthal, diſeaſes of, — — II. 289

+ > Cline, Henry, diſſection of an aneuriſm, — VII 405

Clitoris, cancerous, caſe of, — — II. 115

Clowes, Tho. caſe of hernia, — — X. 72

Cocoa-nut tree deſcribed, — — VIII. 291

Coffee tree, when introduced into Jamaica, — ibid. 246

Colchicum, remarks on its effects in dropſy, — I. 395. II. 297

Cold, experiments on, I. 433; extreme degree of, IV. 205

———, power animals have of producing it, — III. 122

Coleman, William, two caſes of ſcurvy, — II. 117

———, caſe of worms diſcharged through a wound of the abdomen, — — VII. 251

Colic, occaſioned by a piece of bone in the inteſtines, — I. 327

Collin, H. on the effects of lactuca ſylveſtris in dropſy, ibid. 263

Colombo

+ > Cline Mr. Expts to prove that no air exists in the blood in an expansible state. IV. p. 272.

Colombo root, remarks on, — — II. 355

Colon, prolapsus of, — — I. 335

Commentationes Gottingenses, — II. 73, 361. IV. 338

Comus, Sieur, experiments in medical electricity, — V. 58

Concussion of the brain, case of, — II. 169

Consumption, venereal, cured by mercurial frictions, — I. 129

Contagion, febrile, remarks on, — X. 260. XI. 329

Convulsions during parturition, remarks on, — II. 328

Cook, Captain, the healthiness of his crew instanced, III. 48

——, Mr. remarkable case of a fractured scull, — IV. 72

—————— dropsy, — VII. 54

Copland, Peter, on amenorrhœa, — — XI. 230

Copper, poison of, its effects, — — II. 411

——— vessels, a mode of lining them described, ibid.

Cornette, M. chemical essays by, IV. 28, 30. V. 346, 347, 352

Coronary vein, its orifice described, — — II. 35

Cortex Angusturæ. See Angustura.

Coste, Dr. J. F. experiments with opium in lues venerea, IX. 7

Costiveness, case of, — — I. 349

Covey, John, obs. on inoculation, — VII. 180. VIII. 3

———— a disease of the lymphatics, VIII. 136

Cowhage, accounts of, — — VI. 313. VIII. 253

Cowling, Dr. R. case of hemiplegia, — II. 198

Crawford, Dr. A. examination of his theory of heat, — I. 109

———— of the power of animals to generate cold, III. 122

Cribb, W. case of luxation of the thigh, — V. 412

————, on the treatment of hernia, — VI. 259

Crichton, Dr. A. on the astragalus exscapus, — IX. 405

———— lichen islandicus and arnica montana, X. 229

Crinons, account of a disease so called, — III. 289

Croft, Richard, method of reducing the funis, — VII. 38

————, cases of retroverted uterus, — XI. 380

Crotchet, its use in cases of narrow pelvis, — VII. 40

Crusta lactea, obs. on, — — II. 187

Cullen, Dr. W. First Lines of Physic, — VI. 60

Cullum, T. G. case of a tumour between the rectum and bladder, ibid. 315

Cupping, antiquity of, — — III. 266

Cuprum ammoniacum, good effects of, — VII. 187

Cullon, M. anecdotes of, — — V. 318

### D.

Daniel, Dr. Samuel, case of painful menstruation, — V. 183

Darby, John, case of emphysema, — — VIII. 407

Darbey, R. on the use of cod-liver oil in rheumatism, III. 398

David, M. on necrosis, — — ibid. 369

Davidson, Tho. facts relative to small pox, — X. 353

Daubenton, M. remarks on sheep, — — II. 108

Dawson, Dr. Tho. his remedy for sore eyes, — III. 180

Deafneſs, inſtance of, aſcribed to metaſtaſis, —— II. 19

——, uſe of electricity in, —— — XI. 31

Deaſe, W. account of a fatal caſe of ganglion, —— V. 172

Deglutition, impeded, caſes of, —— —— X. 356

Denman, Dr. T. on the ſpontaneous evolution of the fœtus, V. 64, 301

—— uſe of the globe peſſary, — VII. 56

—— account of a diſeaſe obſerved in infants, XI. 374

Diabetes, facts and obſ. relative to, I. 89. IX. 286, 290, 294, XI. 221

Diarrhœa cured by verbaſcum, I. 96; by ipecacuanha, VI. 422

Dickſon, Dr. Stephen, on pemphigus, —— IX. 309

Diet, remarks on, —— —— I. 411

Digeſtion, remarks on, —— —— III. 309

Digitalis purpurea, its effects, II. 415. VI. 55, 145, 298. VII. 191

Dijon, Memoirs of the Academy at, IV. 379. V. 133. XI. 265

Dillon, P. on the cure of fiſtula in ano by cauſtic, — V. 392

Dimſdale, Baron, his tracts on inoculation, —— II. 145

Dobſon, Dr. M. account of the harmattan, —— *ibid.* 194

——, on the effect of a reſuſcitated ſalivation, VI. 419

Douglas, Dr. Andrew, caſe of a ruptured uterus, VI. 91. X. 100

—— ſingular cough, —— VI. 416

Dropſy, facts and obſervations relative to, I. 91, 263, 266. V. 106, 315. VI. 213. VII. 54, 157, 189. VIII. 233, 248, 254. X. 149

——, cured by the lactuca ſylveſtris, — — I. 263

—— blue vitriol, —— I. 266. X. 149

——, effects of the digitalis purpurea in. See Digitalis.

—— tobacco in, —— — VI. 185

——, contents of the medullary cells in, — VII. 157

Drummond, Dr. Alex. Monro, anecdotes of, — III. 323

Dryander, Jonas, on the Benjamin tree of Sumatra, — IX. 80

Dublin Lying-in Hoſpital, abſtract of its regiſtry, — *ibid.* 199

Dubourg, J. Barbeu, anecdotes of, —— IV. 40

Dujardin, M. hiſtory of ſurgery, —— — III. 260

Duncan, Dr. And. letter to Dr. Jones, —— *ibid.* 104

—— abridgement of Hoffman's practice of phyſic, V. 160

Dundas, David, caſe of hydrophobia, —— VIII. 156

Dyſentery, obſ. on, II. 86, 211, 379. III. 189. V. 137, 375. VII. 337

Dyſury, chronic, remarks on, —— — IV. 69

——, effects of tobacco in, —— VI. 189

### E.

Ears, their conſent with the lungs, —— I. 179

Eau de luce, method of preparing it, —— *ibid.* 193

Elbow joint, ſingular diſeaſe of, —— —— *ibid.* 329

——, new mode of operating in diſeaſes of, IV. 273. XI. 29

Electricity, medical, remarks relative to, I. 246, 374. VII. 10, 247, 115, 141. XI. 31

——, its efficacy in retention of urine, —— VII. 10

—— a caſe of diſeaſed teſticle, —— *ibid.* 247

—— a painful affection of the face, *ibid.* 115

——, recommended in the cataract, —— *ibid.* 141

Electricity, its effects in deafness, — — XI. 31

———————— a case of wry neck, — ibid. 385

Electrophorus, perpetual, account of, ——— II. 29

Elliott, Dr. John, case of obstinate costiveness, — I. 349

————————, his physiological essays, ——— I. 438

————————, on mineral waters, ——— II. 320

————————, his edition of Dr. Fothergill's works, ibid. 417

————————, elements of natural philosophy, — III 59

Else, Joseph, account of an edition of his works, — ibid. 380

Emmenagogues, remarks on the inefficacy of, ——— I. 147

Enaux, M. on luxation of the pelvis, ——— XI. 265

Epilepsy, facts and observations relative to, I. 428. IV. 76. V. 58, 361. VI. 321. VII. 52

Evans, Dr. John, case of a ganglion, ——— VIII. 154

Ewer, Dr. J. account of a new species of bark, ——— X. 154

Eye, cancerous, successfully extirpated, ——— III. 6

——, dropsy of, case of, ——— ——— I. 346

——, purulent, remarks on, ——— ——— ibid. 115

——, orbit of, tumour extracted from, ——— III. 6

——, sore, remedy for, ——— ——— ibid. 180

Experience in physic, treatise on, ——— ——— IV. 9

F.

Fabbroni, J. account of an experiment in hydrophobia, IX. 69

Face, painful affection of, cured by electricity, — VII. 115

Fæces, hardened, cases of, ——— VI. 355. VII. 26

Falconer, Dr. W. on the influence of climate, ——— I. 409

Fearon, Henry, method of amputating a breast, — IV. 4[illegible]6

Fecundation of plants, discovery relative to, — II. 210

Fever, epidemic, accounts of, ——— IV. 53. X. 117

——, intermittent, obs. relative to, I. 2, 389. II. 267, 305 VII. 192. VIII 191. IX. 48. X. 154, 158. XI. 38

——, miliary, ————————, — — I. 168

——, puerperal, ————————, — I. 9, 169, 408. III. 411

——, putrid, ————————, — I. 384. III. 45, 268

——, remitting, of the West Indies, remarks on, — II. 253

——, use of cold bathing in, ——— ——— VII. 109

——, exanthematous, observations on, ——— III. 362

Filkin, Mr. on the extirpation of the knee joint, — XI. 27

Fire, observations on, ——— — I. 312. II. 63

——, St. Anthony's, inquiries relative to, ——— III. 3[illegible]3

Fisher, Joshua, case of tumour in the abdomen, — VII. 287

Fistula in ano, case of, I. 320; instrument for, XI. 228

——— lachrymalis, remarks on, ——— II. 77, 245

————————, case of, ——— ——— II 174

——— in perinæo, cases of, — — I. 379. IX. 378

Flooding, uterine. See Hæmorrhage.

Fluor albus, remarks on, ——— ——— I. 151

Fœtus, misplacement of the viscera in one, ——— I. 205

——, extra-uterine, instances of, V. 396. VI. 52. VIII. 147, 321, 328

Fœtus, head of one, feparated in the uterus, — I. 384
——, in utero, infected with fmall pox, — II. 204
——, fpontaneous evolution of, inftances of, V. 64, 301. VI. 371
Fontana, Abbé, on poifons, — V. 1, 417
Ford, Edward, on the hydrophthalmia, — I. 346
——, account of an operation on a broken arm, II. 46
—— ulceration of the bladder, III. 80
—— a ftrangulated hernia, — VI. 118
——, cafes of wounds of the head, — VIII. 411
——, of the fpontaneous cure of aneurifms, IX. 142
——, on hydrocephalus internus, — XI. 56
——, account of a wound of the ulnar artery, ibid. 357
—— calculus extracted from a cyft, ibid. 362
Fofter, Dr. Edward, treatife on midwifery, — I. 402
Fothergill, Dr. John, account of his works, — II. 417
——, biographical anecdotes of, — IV. 178
—— Ant. hints on fufpended animation, III. 185
——, on the puerperal fever, — ibid. 411
Foundlings at Aix, mortality among them inftanced, II. 66
Fowler, Dr. Thomas, on the effects of tobacco in dropfy, VI. 185
—— arfenic in intermittents, VII. 192
Fox glove. See Digitalis.
Fractured limbs, machines for, defcribed, — I. 239. III. 334
Fragility of the bones, cafes of, — IV. 309. VI. 288
Franco, P. his method of cutting for the ftone, — X. 166
Frederick II. King of Pruffia, account of his laft illnefs, IX. 329
Friccius, Melchior, his praife of arfenic in intermittents, VII. 194
Fritfe, F. A. cafe of Cæfarean fection, — XI. 146
Funis, method of reducing it, — — VII. 38
Furor uterinus, how treated by Mofchion, — III. 343
Furunculus, cafe of, — — I. 332

G.

Gall bladder, cafe in which it was totally effaced, — IV. 44
—— ftones, folvent of, — — V. 126, 134
Ganglion, fatal cafe of, — — V. 172
——, cafe of, fuccefsfully treated, — VIII. 154
Gangrene, obfervations on, — V. 75, 190. VI. 131. XI. 167
Gapper, Edm. Pitts, cafe of phthifis, — XI. 388
Garlick, William, account of two diffections, — V. 186
Garthfhore, Dr. Maxwell, on extra-uterine cafes, VIII. 328
—— numerous births, — X. 83
Gaftric juice, its effects as a topical application, — VI. 405
——, recommended in the bite of a rabid animal, X. 306
Gaubius, J. D. anecdotes of, — I. 137. V. 119
Geach, Francis, on an epidemic dyfentery, — II. 379
Gellert, C. E. metallurgical chemiftry, — I. 140
Generation, fact relative to, — — II. 109
Gilby, Dr. W. cafe of wry neck cured by electricity, XI. 385
Gillefpie, Leonard, on the putrid ulcer, — VI. 373
—— health of feamen, — VIII. 113

 Girtanner,

Girtanner, Dr. C. on the venereal difeafe, — IX. 427. X. 336
Gleets, how to be treated, — — II. 244
Gonorrhœa, obfervations relative to, — II. 233. III. 214
Goodwin, W. cafe of foftnefs of the bones, VI. 288. VIII. 67
———— an encyfted tumour, — VI. 292
Gottingen, Comment. of the R. Society at, II. 73, 361. IV. 338
Gout, obfervations relative to, — — I. 199, 210
——, relieved by the ufe of cicuta, — II. 410. V. 86
——, medullary, inftance of a difeafe fo called, — IV. 309
——, in the ftomach, relieved by æther, — VI. 53, 341, 348
Grant, Alex on the ufe of opium in morbid irritability, VI. 5, 130
——, Geo. cafe of abfcefs of the belly, — XI. 138
Gratiola, recommended in venereal caries, — II. 222
Graves, Dr. Robert, cafe of a fcirrhous ftomach, — XI. 343
Green, J. L. defcription of a lufus naturæ, — IV. 403
Gregory, Jac. confpectus medicinæ theoreticæ, — V. 382
Grieve, Dr. John, his account of koumifs, — X. 197
Grimfton, John, cafe of a fractured fcull, — ibid. 277
Guaiacum, gum, the tree that produces it, defcribed, VIII. 258
Gum, elaftic, experiments relative to, — III. 221. IV. 108
————, generical character of the tree that yields it, VI. 315
—— tree, accounts of different fpecies of, VIII. 270. XI. 308

H.

Hæmorrhage, uterine, obf. relative to, I. 59, 148, 155. II. 122
————, fatal, from the anus, — I. 382
————, from the lungs, inftance of, — VI. 252
Haen, Anton. de, opera pofthuma, — II. 390. IV. 49
Hahn, J. D. letters from, — III. 313, 431
Haighton, John, cafe of hydrophobia, — VI. 361
Hair, maffes of, found in the ftomach, — IV. 361
—— powder, differtation on, — — III. 217
Haire, Launcelot, remarks on amputation, — VII. 377
Hale, John, cafe of fracture of the fternum, — VIII. 391
Hall, John, on the contents of the medullary cells in dropfy, VII. 157
——, Richard, cafe of a fcirrhus of the fcrotum, VIII. 81
Haller, Albert Von, anecdotes of, — II. 341
Halliday, Dr. W. his letter on the red bark, — IV. 416
Halloran, Silvefter O', obfervations on the cataract, X. 395
Hamilton, Alexander, treatife on midwifery, — I. 145
————, Dr. Robert, (of Ipfwich) cafe of a difeafed tefticle, IV. 172
———————— on the effects of opium in gangrene, — — V. 75, 190
———————— a cafe of mollities offium, VI. 291
———————— the hydrophobia, VII. 89
———————— a cafe of worms difcharged thro' the navel, — — ibid. 372
————, (of Lynn) obf. on the mumps, XI. 190
Hand torn off by a mill, cafe of, — I. 325
Harmattan, account of — — II. 194

Hawes

Hawes, Dr. W. addrefs to the King and Parliament, — III. 184
——, letter from, — — IV. 95
Hayes, Thomas, on intermittents, — II. 267
Haygarth, Dr. John, on the prevention of fmall pox, VI. 206
—— hydrophobia, X. 295
Head, cafes of wounds of, I. 323, 324, 325. II. 13, 17, 18, 175, 176. III. 2, 279. IV. 72. V. 278. VII. 152. VIII. 411. X. 277
——, peculiar tumour on, — — III. 4, 146
Heart, obfervations on the rupture of, — IX. 156
——, malconformation of, inftances of, VI. 407, 432, 434, 436. VII. 280, 284
——, remarkable difeafe of, — — X. 341
——, ulcer of, — — IV. 358
Heat, animal, obfervations and queries relative to, — VII. 169
Hectic fever, remarks on, — — II. 280
Hellebore, remarks relative to, — — I. 190
Hemiplegia, cafes of, — — I. 323. II. 198
Hemlock, its efficacy in the gout, — II. 410. V. 86
——, dropwort, poifonous effects of, — II. 40. V. 192
—— water, ——, — I. 48
Henbane, obfervations relative to, — I. 338. II. 295
Henry, Thomas, method of preferving water frefh at fea, II. 135
——, of the medicinal effects of magnetifm, III. 303
——, remarks on fermentation, — IV. 208
——, memoirs of M. de Haller, — *ibid.* 217
——, experiments on latent heat, — V. 89
——, tranflation of M. Lavoifier's effays, *ibid.* 159
Hepatitis, cafes and obfervations relative to, I. 391. VIII. 44. X. 142
Herbiniaux, M. on laborious deliveries, — III. 161, 242, 441
Hermaphrodites, inftances of what are fo called, III. 44. IV. 403
Herpes, remarks on, — — I. 94, 240
Hernia, obf. relative to, II. 13, 18, 126, 169, 170. III. 12, 273. VI. 118, 259. X. 72
——, humoralis, cafe of, — — II. 177
Heyfham, Dr. J. on the jail fever at Carlifle, — III. 268
Hints on difeafes that are not cured, — — I. 364
Hippocrates, his defcription of an epidemic catarrh, IV. 434
Hird, Dr. William, anecdotes of, — — III. 325
Hoffman, his anodyne mineral liquor, how prepared, VI. 351
Holman, Charles, cafe of phthifis unexpectedly relieved, VII. 120
Home, Dr. Francis, clinical experiments, — I. 1, 85
——, Everard, on the popliteal aneurifm, VII. 391. VIII. 126
——, Robert, on folvents, — — IV. 382
Homer, his medical knowledge extolled, — III. 264
Hope, Dr. John, defcription of a plant that yields affafœtida, VII. 68
Hopfon, Dr. C. R. his theory of fire, — — II. 63
Horn diftemper, in cattle, defcribed, — — VII. 305
Houlfton, Dr. Tho. cafe of a boy poifoned by hemlock dropwort, II. 40
——, of a mineral fpring at Wigan, — V. 311
——, on the ufe of mercury in dyfentery, *ibid.* 374
——, letter to, from a phyfician in Italy, *ibid.* 419

Houlston, Dr. Tho. case of a man who swallowed corrosive sublimate, ——— ——— VI 271

———, experiments with variolous matter, VII. 7

———, William, case of injury of the brain, — V. 292

Houndsfield, Geo. on the cure of diseased testes by electricity, VII. 247

Houstouni reliquiæ, ——— ——— V. 253

Howard, John, on the medical properties of mercury, III. 170

——— cure of hydrocele by seton, — V. 61

Hughe , T. case of a cancer of the breast, ——— X. 40

Hulke, William, case of remarkable spasmodic affection, V. 389

Hunckzowsky, J. of the use of green walnut shells in ulcers, X. 319

Hunter, Dr. William, introductory lectures, ——— VI. 190

———, cases and remarks by, *ibid.* 43, 433, 437

———, John, on the hearing of fish, ——— IV. 229

———, case of hydrophobia, ——— VII. 89

———, of diseases produced by transplanting teeth, *ibid* 205

———, his operation for the popliteal aneurism described, *ibid.* 391

———, remarks on mollities ossium, — VIII. 70

———, on the effect of extirpating one ovarium, IX. 71

Hurricane, effects of one in Jamaica on the sick, — I. 133

Hydatids, description of, ——— ——— X. 77

——— voided by the mouth, cases of, — VI. 139, 293

——— with the urine, ——— ——— I. 125

Hydrocele, facts and observations relative to, II. 174. III. 16, 144, 381. VIII. 119 IX. 109

Hydrocephalus internus, obs. relative to, I. 357, 358, 424. III. 402. IV. 78, 193. V. 354. VI. 113, 214. IX. 122. XI. 56

———, as it occurs in maniacal cases, described, VI. 159

Hydrophobia, facts and obs. relative to, I. 370. II. 132, 206, 272. III. 23, 92, 288, 307, 312. V. 85, 220, 286, 314, 326, 421. VI. 361. VII. 81, 89. VIII. 156. IX. 69. X. 292, 295. XI. 143.

———, spontaneous, cases of, ——— IX. 256, 267

Hydrophthalmia, case of, ——— ——— I. 346

Hypochondriasis, remarks on, ——— — *ibid* 398

Hysteria, cured by the bark, ——— ——— *ibid.* 383

### J.

Jackson, Dr. Robert, on the lunar influence in fevers, VIII. 25, 300

——— treatment of gun-shot wounds, XI. 363

Jacob, Edward, case of luxation of the os femoris, V. 312

——— extra-uterine fœtus, — VIII. 147

Jalap, substitutes for it described, — V. 129. VIII. 271

Jamaica, obs. on the climate and diseases of, II. 63. III. 73

———, medicinal plants of, described, ——— VIII. 217

James's powder said to have excited ptyalism, — XI. 297

Janssen, J. F. on the effects of fixed air in putrid fever, IV. 74

Ice water, good effects of, in scirrhus, ——— *ibid.* 350

Jebb, Dr. John, on the palsy of the lower limbs, — III. 372

Jenner, J. C. caſe of excreſcence in the urethra, — VII. 160

——, account of a general inoculation at Painſwick, *ib.* 163

—— —— the efficacy of arſenic in intermittents, IX. 48

Impotency ſaid to be frequent in Algiers, — XI. 144

Inflammable air known to Van Helmont, — II 424

——, obſervations relative to, — IV. 6

Influenza. See Catarrh.

Inoculation, obſ. relative to, I. 429. IV. 438. VII. 7, 63, 66, 163, 180. X. 270, 3[illegible]3

——, early practice of, — — XI. 141, 234

Intermittents. See Fever.

Intoxication, exceſſive, caſe of, — I. 396

Johnſon, James, caſes by, — VI. 293, 295, 354, 355

Johnſtone, Dr. J. caſe of a gun-ſhot wound, — VII. 135

——, account of a remarkable effect of opium, *ibid.* 136

Jones, William, caſe of a fractured ſcull, — V. 278

Ipecacuanha, ſubſtitutes for it, deſcribed, *ibid.* 130. VIII. 228

——, two ſpecies of, found in Brazil, — IX. 69

Iris, its ſtructure explained, — — X. 400

Iron, remarks on ſome ſaline combinations of, — IV. 27

Irving, Dr. R. on the ſolvent power of magneſia, VII. 419

Iſinglaſs, how prepared, — — I. 25

Itch, new remedy for it deſcribed, — — V. 350

——, inſect found in, deſcribed, — — IX. 28

——, two ſpecies of, diſtinguiſhed by the ancients, — *ibid.* 38

Juſſieu, Bernard de, anecdotes of, — — II. 344

——, Joſeph de, anecdotes of, — — V. 337

### K.

Keir, Dr. William, caſe of a fatal vomiting, — VII. 72

Keyſer's pills, baneful effects of, — — I. 206

Kidney, enlargement of, inſtances of, I. 388. VI. 427, 428

Kinglake, Robert, caſe of dilatation of the femoral artery, VIII. 385

—— —— a diſeaſe of the heart, — X. 341

Kippis, Rev. Dr. his life of Sir John Pringle, — V. 140

Kirkland, Dr. Tho. on the preſent ſtate of medical ſurgery, *ibid.* 384

—— —— uſe and abuſe of mercury, VII. 1

Kirwan, R. on the ſpecific gravities of ſalts, II. 192. III. 5. V. 39

Kite, Charles, of a remarkable nervous affection, — III. 3[illegible]0

——, caſe of paralyſis ſucceſsfully treated, *ibid.* 405

—— the uſe of electricity in the cataract, VII. 141

——, caſes of obſtruction of the bowels, VIII. 164

Knee joint, inſtances of its being extirpated, IV. 2[illegible]8. XI. 22, 27

Knight, Francis, effects of a large doſe of ſugar of lead, III. 286

Koelpin, A. B. on the rhododendron chryſanthum, — I 226

Koumiſs, its medicinal properties, — — X. 197

Krzowitz, W. Trnka de, de febre hectica, — VI. 81

Labour,

### L.

Labour, theory of, ——— ——— IV. 355

Lactuca sylvestris, its efficacy in dropsy, ——— I. 263

Languedoc, account of an epidemic fever in that province, IV. 53

Lapland, Swedish, population of, ——— — III. 22

Lavoisier, M. chemical and philosophical essays by, II. 106, 109, 178, 179, 181, 184, 185, 228. IV. 35, 235, 414

Laurel water, its effects described, ——— I. 343. XI. 160

Leather, Russian, manner of preparing it, ——— I. 23

Ledum buxifolium, description of, ——— — II. 33

——— palustre, said to have been efficacious in leprosy, ibid. 137

Lee, Dr. John, narrative of a singular gouty case, — III. 107

Leech, obs. on its nat. hist. and medicinal uses, I. 237, 399. III. 139

Lefevre, M. conjectures concerning his remedy for the gout, I. 201

Leg, artificial, account of one ingeniously contrived, IX. 254

Lentin, L. F. Benj. de morbis Clausthaliensium, ——— II. 289

Leonardo da Vinci, his anatomical skill, ——— VI. 191

Leprosy, a species of, in the Crimea, described, ——— I. 28

———, account of, at Martigues, — — III. 289

Lever, of Roonhuysen, description of, — — ibid. 165

Lewis, Dr. William, account of his manuscripts, — II. 50

———, abridgement of Hoffman's pract. of physic, V. 160

———, exp. hist. of the materia medica, VI. 89

Lichen islandicus, observations on its medicinal effects, X. 229

Lieutaud, M. some account of, — I. 138, 279. V. 117

Lightning, fatal effects of, ——— ——— II. 192

Lime water recommended in ulcers, ——— II. 16

——— for impregnating water at sea, ibid. 135

Lind, Dr. James, on the effects of vitriolic æther in the gout, VI. 53

——— mercury in inflammation and dysentery, ——— ——— VIII. 43

——— influence of the moon in fevers, ibid. 145

——— tænia hydatigena, ——— X. 76

——— John, on the efficacy of flowers of zinc in epilepsy, VII. 52

Linnæus, the elder, view of his life and writings, III. 29. IV. 26

———, English transl. of his Systema Vegetabilium, V. 107

——— younger, Supplementum Plantarum, ——— V. 256

———, some account of, ——— ibid. 329

Lip, upper, swelling of, described as a symptom of worms, I. 95

Lithontriptics, remarks on, ——— I. 97. IV. 382

Lithotomy, observations relative to, — III. 16. XI. 237

———, at two different times, account of, IV. 242. X. 162

Liver, facts and obs. relative to the diseases of, I. 390. II. 230. IV. 44

———, abscess of, after a blow, ——— ——— VII. 22

———, diseased, attended with pain in the calf of the leg, II. 291

Loftie, William, exfoliation of part of the upper jaw, IX. 57

———, case of a scirrhous stomach, — XI. 17

Logwood, observations relative to, ——— VIII. 261

London, comparative table of the population of, — III. 120

Lorry, M. cases and observations by, — IV. 43, 371

Lucas, James, remarks on cataracts, — VI. 429

———— amputation, VII. 225. VIII. 142. IX. 223

———— elastic bandages, — IX. 44

———— febrile contagion, X. 260. XI. 329

————, case of a puncture of the bladder, — XI. 109

————, cases of retention of urine, XI. 115, 118, 121

————, remarks on diseases of the bladder, — *ibid.* 123

————, account of singular effects of music on a patient, *ib.* 125

———— uncommon symptoms after the measles, *ibid.* 325

Lues venerea, of the treatment of, in children, — I. 388

————, observations relative to, II. 217, 233, 357. VII. 328

————, effects of the astragalus exscapus in, — IX. 405

Lumbago, remedy for, — — — I. 87

Lungs, case of a wound through, — — *ibid.* 328

### M.

Macbride, Dr. David, some account of, — IV. 39

Mackenzie, Dr. Colin, letters on midwifery subjects, II. 421

Macqueen, Dr. Malcolm, on angina pectoris, — V. 162

Madder, root of, its effects in amenorrhoea, I. 93. XI. 230

Magnesia, account of Prof. Bergman's dissertation on, I. 83

————, its effects as a solvent of bark asserted, VII. 254. VIII. 75

———— controverted, VII. 419

————, calcined, experiments on, — III. 96

Magnet, its medicinal effects described, III. 158, 303. V. 364

Magnetism, animal, reports of the French Academicians relative to, — — V. 266. VI. 103

Maguire, Laur. White, case of a lumbar abscess, VII. 14

Mahogany bark, its medicinal properties, — VIII. 286

Majault, M. strictures on several chemical preparations, II. 315

Malouin, M. some account of, — — IV. 24

Malt, infusion of, its good effects in pyuria, — VI. 295

Malta, answer to queries from, by the Med. Soc. at Paris, II. 214

Malvern water, account of, — II. 346

Manchester, Philos. Soc. of, account of, — III. 91

Mania, cases of, — II. 38, 198, 292. VI. 159

Maret, M. (the elder) some account of, — I. 371

———— (the younger) on the construction of hospitals, IV. 380

Marseilles, population and diseases of, — IV. 369

Martin, R. on the external senses, — III. 20

Masturbation, remarkable instance of, — IV. 302

May, Dr. William, on the treatment of phthisis, IX. 268, XI. 255

————, account of an epidemic catarrh, IX. 342

———— fever, X. 117. XI. 211

Measles, treatment of with blisters, — III. 183

————, uncommon symptoms after, described, XI. 325

————, fact relative to the contagion of, — *ibid.* 329

Mediastinum, abscess of, — — II. 405

Medical obf. and inquiries, abftract of, VI. 211, 321, 414

Medicines, remarks on the operation of, IV. 371. XI. 287

Melæna, remark on, —— —— I. 10

Membrana decidua, Aretæus and Galen faid to allude to it, III. 341

———— pupillaris, remarks on, —— IV. 359

Menfes, final ceffation of, with what fmyptoms attended, I. 150, 175

————, remedies in obftruction of, I. 92, 147, VIII. 412. IX. 230

Menftruation, painful, how to obviate, —— I. 148

————————, cafe of, — —— V. 183

Mercury, treatife on the medical properties of, III. 170

————, crude, curious effects of, —— VIII. 85

————, congelation of, account of, IV. 205. VI. 227, 230, 233

————, its effects in dyfentery and inflammation, V. 375. VIII. 43

———————— impeded deglutition, —— X. 356

———————— fpafm of the bladder, — ibid 360

————, fmall dofes of, recommended in fyphilis, VIII. 1

————, ufe of, prohibited in the Italian hofpitals, II. 217

Mefembryanthemum cryftallinum, its med. properties, IX. 100

Metaftafis, remarks on, —— —— I. 178

Meteorology, remarks relative to, — — IV. 341

Mezereon, bark of recommended for iffues, — II. 273

Michaelis, Dr. F. on the ftate of fcorbutic patients, ibid. 127

———————— hydrophobia, — V. 286

———————— decuffation of the optic nerves, ibid. 289

Michell, Jan. Peterfon, de fynchondrotomia pubis, IV. 374

Milk, human, obfervations on the properties of, XI. 71

Millepedes, analyfis of, ———— ———— II 355

Millington, L. account of the cultivation of aloes, VIII. 422

Milman, Dr. Francis, on the origin of fcurvy, III. 45

Mills, S. G. cafe of a lady who fwallowed a pin, VI. 36

Monro, Dr. Alex. (the elder) account of his life and works, I. 297

———————— (the younger) on the nervous fyftem, IV. 113

Monfters, defcriptions of, —— IV. 4. X. 422. XI. 339

Moon, obfervations on its fuppofed influence on the human body, VI. 395. VIII. 25, 145, 300

Moore, J. on diminifhing pain in furgical operations, V. 369

Morgan, W. obf. on Dr. Crawford's theory of heat, I. 109

Mortality, obfervations on bills of, —— IV. 2. VII. 316

Morton, Dr. D. cafe of a Cæfarean operation —— VII. 61

Mofeley, Dr. J. cafe of hydrocephalus, ———— VI. 113

————, Benjamin, on the dyfentery, ———— II. 86

Moffes, their fexual fyftem difcovered, —— IV. 437

Moufet, Tho. his defcription of the itch infect, — IX. 29

Moyle, Richard, cafes and remarks by, VI. 52, 252, 257

Mules, vegetable, remarks on, ———— II. 33

Mumps, account of a difeafe fo called, —— XI. 190

Mungo root, account of —— —— II. 354

Munning, J. cafe of a curvature of the fpine, — VI. 358

Murray, J. And. botanical remarks by, II. 71, 361. IV. 338

————, Adolphus, de lue venerea, —— II. 217

———————— fenfibilitate offium, — III. 222

Mufcles, obfervations on the noxious effects of, ibid. 134, 135

Mufhrooms,

Mushrooms, noxious qualities of, —— ibid. 362

———, precautions of the Chinese with respect to, II. 32

Music, instances of its medicinal effects, I. 183, II. 425, XI. 125

Musk, recommended in a particular species of gangrene, XI. 169

Mynors, R. remarks on a case of fractured skull, V. 284

N.

Napellus cæruleus, its effects in rheumatism, I. 340, II. 39

Natron, of Russia, remarks on, — —— II. 32

Navel, cautions with respect to the treatment of, in infants, VII. 376

Necrosis, observations relative to, III. 369. VII. 263

Nerves, treatises on the diseases of, I. 32, 98, 174. II. 347, 425, 426

———, of the 2d and 3d cervical pairs, described, II. 105

———, reproduction of, experiments relative to, V. 23

———, structure of, as examined with a microscope, ibid. 25

———, optic, decussation of, in quadrupeds, — ibid. 289

Newman, Jer. Whitaker, on solvents, —— II. 222

Nobleville, M. Louis Arnold de, some account of, IV. 38

Nux vomica, recommended in dysentery, — III. 189

O.

Odier, Dr. Lewis, on the magistery of Bismuth, VII. 321

O Donnel, John, case of a man covered with wens, VI. 33

Oenanthe crocata, poisonous effects of, —— II. 40. V. 192

Oesophagus, abscess of, ——— —— III. 157

———, instances of schirrous affection of, IV. 45, 46

Oliphant, Isaac, case of an abscess of the liver, — VII. 22

——— hardened fæces, —— ibid. 26

——— incontinence of urine, ibid. 416

Oliver, Dr. W. effects of camphor in mania, — VI. 120

Omentum, suppuration of, — — I. 241, 336

———, dropsy of, —— —— ibid. 423

Ophthalmia, remarks on, — —— — ibid. 115

Opium, use of in intermittents, condemned, — ibid. 136

———, its effects in syphilis, III. 221. IV. 419. IX. 7

——— obviating morbid irritability, VI. 5, 130

———, new method of preparing an extract of, III. 283

———, observations on the analysis of —— ibid. 360

———, remarkable effect of, ——— VII. 136

Orichalcum, of the ancients, what, —— III. 141

Osborn, Dr. William, on laborious parturition, IV. 135

Ovarium, singular tumour of, — —— I. 394

———, curious appearance of, on dissection, ibid. 203, X. 322

———, extirpation of one, effects of, —— IX. 71

Oxford, school of physic at, —— —— III. 422

P.

Pain, in surgical operations, mode of preventing, V. 369

Palfyn, Jean, inscription to the memory of, — ibid. 330

Pallas, P. S. his travels, —— —— I. 20, 185

Palsy, experiments in cases of, — —— ibid. 85

Palfy, of the lower limbs, remarks on, III. 225, 372, 405
Paré, Amb. referred to on the fubject of tranfplanting teeth, X. 243
Pareira Brava, its medicinal properties, — VIII. 241
Paris, memoirs of the Royal Academy of Sciences at, II. 103, 178, 227. IV. 24. V. 337. VI. 179, 305
——— Royal Medical Society at, III. 149, 273, 353. IV. 38, 148, 238, 358. V. 113, 248, 348
Park, H. on a new mode of operating in difeafes of the knee and elbow, — — IV. 273. XI. 22
———, obfervations relative to, VII. 226, 383
Paronychia, a particular kind of, defcribed, — III. 17
Parotids, extirpation of, — — ibid. 8
Partington, Miles, cafe of amaurofis, — — IX. 389
Pafcoe, James, cafe of a tumour of the leg, — IV. 141
Patten, S. cafes of impeded deglutition, — X. 356
Payne, Thomas, cafe of a wound in the throat, — VI. 28
Pearfon, Dr. G. of phofphorated foda, — IX. 393
———, John, on animal heat, — VII. 169
Pelvis, obfervations on the luxation of the bones of, XI. 265
Pemphigus, obfervations relative to, IX. 309. X. 361. XI. 234
Percival, Dr. T. on the ufe of cod liver oil in rheumatifm, III. 392
———, mifcellaneous facts and obfervations, IV. 56
———, on the rabies canina, — X. 295
——— operation of medicines, XI. 287
Perfect, William, cafes in midwifery, II. 419. IV. 216
Pericardium, account of an extravafation of blood into it, VI. 211
Perinæum, cafe of tumour in, after delivery, — IX. 119
Peterfburgh, memoirs of the Imperial Academy at, II. 26. V. 129
Peyrilhe, M. hiftory of furgery, — — III. 337
Pharmacopeia Brunfvicenfis, — — I. 189
——— Edinenfis — — V. 44
——— Genevenfis, — — II. 142
——— Londinenfis, — — IX. 88
——— Wirtenbergica, — — VII. 108
Philip, Governor, his account of tipioca, — IX. 69
Phlogifton, differt. on the quantity of in different metals, II. 141
———, experiments relative to, — III. 88. V. 238
Phofphorus, effects of in fevers, — — I. 208
———, remarks on the combuftion of, — II. 106
——— acid of, — ibid. 183
Phthifis, obf. on, I. 9. III. 385. VII. 120. IX. 268. XI. 255, 388
Phyficians, obf. on the ftate of, among the ancients, IV. 110
Pins, fwallowed, inftances of, — I. 334. VI. 36, 400
Placenta, preternatural retention of, — — II. 38
———, of a fingular ftructure, — — ibid.
Plague, obfervations relative to, III. 329. IV. 364. V. 330, 331
Platina, obfervations relative to, — III. 308. V. 344
Poifons, remarks on, I. 38, 336. II. 40, 65, 411. V. 1, 193, 374, 417, 419
Polypus, bronchial, cafe of, — — VI. 252
———, of the heart, remarks on, — — ibid. 225

Polypus, of the nose, obs. relative to, — I. 321. III. 7

——— uterus, treatise on, —— — III. 242

Portal, M. obs. on the rupture of the heart, — IX. 156

——— diseases of the liver, —— II. 230

Pott, J. H. (of Berlin) some account of, ——— I. 137

——, Percival, on the palsy of the lower limbs, III. 225

Pouteau, M. account of his posthumous works, — IV. 349

Powell, J. case of hydatids voided by the mouth, VI. 139

Pregnancy, obs. relative to, — — I. 152, 154, 405

Price, Dr. J. experiments on mercury, silver, and gold, III. 328

Priestley, Dr. Jos. exper. on air and other subjects, II. 1, 94. V. 238

Pringle, Sir John, anecdotes of, —— —— V. 140

Prognostic in acute diseases, observations on, —— III. 257

Psorophthalmy, remarks on, —— —— I. 115

Ptosis, or fall of the eye lids, cause of, ——— IV. 340

Pubis, separation of the bones of, in parturition, III. 14, 278

——, symphysis, section of, facts and obs. relative to, I, 72, II. 22. III. 311, 366. IV. 374. V. 313, 416. VIII. 34, 318. XI. 46

Pui, Simon de, de homine dextro et sinistro, —— IV. 52

Pulteney, Dr. R. on the effects of hemlock dropwort, V. 192

———, view of the writings of Linnæus, III. 29

Pus, observations on the production of, — XI. 264

Putridity, in diseases, a vague term, — — III. 46

Pylorus, schirrous, case of, —— —— IV. 93

Q.

Quassia Simarouba, botanical and medical account of, XI. 91

R.

Rabies Canina. See Hydrophobia.

Rattle snake, facts relative to its poison, V. 14. X, 310, 312

Razoux, Joh. de Cicuta, —— —— —— II. 293

Rectum, instance of its termination in the bladder, — III. 26

——, obs. on the puncture of the bladder through it, XI. 349

Reeve, Tho. case of a tumour, *in perinæo*, after delivery, IX. 119

Reichel, Baron, his account of a monster, —— X. 422

Reid, Dr. T. on phthisis pulmonalis, —— — III. 38[illegible]

Renny, G. on the venereal disease, — — — IV. 245

Rheumatism, remedies for, — — I. 86, 87, 226. III. 392

Rhus radicans, account of its properties, — — IX. 426

Ribs, motion of, in respiration, —— —— IV. 32, 3[illegible]

——, fractured, case of, —— —— XI. 130

Richter, Aug. Gottlieb, surgical remarks by, II. 77, 363. IV. 339

Ring, John, cases of Pemphigus, — — — XI. 234

Riollay, Dr. F. on the doctrines of Hippocrates, — VI. 86

Roberts, B. cases of women inoculated during pregnancy, V. 399

Robins, John, case of scurvy, —— — — II. 120

Rodbard, John, case of a rupture of the tendo achillis, VIII. 304

Rollo, Dr. John, on the diseases of St. Lucia, — — II. 298

——— effects of drinking spirits, VII. 33

Rosa, Cav. Michela, letters on physiological subjects, — IV. 267

Rose wood, its essential oil commended, — VIII. 222

Roux, M. his edition of Neumann's chemistry, —— II. 69

——, M. le, on the hydrophobia, —— —— VII. 81

Roy, Charles le, on prognostics, —— —— III. 257

Rumsey, Henry, account of an epidemic sore throat, X. 7

Rush, Dr. Benj. on the diseases of the American army, VII. 76

———— Tetanus, —— —— ibid. 424

———— Cancers, —— —— VIII. 89

Russel, James, case of hydrophobia, —— —— IX. 256

Ryan, Dr. Dennis, remarks on remitting fevers, II. 253. III. 63

————, of the Bath water in Jamaica, —— II. 413

————, account of his death, —— III. 211

Rye, smutty, its effects, —— —— —— III. 353, 361

### S.

Sabadilla, seed of, a remedy for the tænia, —— II. 175

Sago Palm, described, — — VIII. 295

Saltpetre, now prepared at Helsingfors, —— — III. 22

Samoilowitz, D. on the cure of the plague, —— ibid. 329

———— inoculation of the plague, V. 330, 331

Sanchez, Antonio Nunes Ribeiro, biographical anecdotes of, V. 92

————, on the Russian baths, ibid. 353

Sanden, Dr. T. on the treatment of rheumatism, — III. 438

————, on some symptoms of fever, —— IV. 289

Saponaria officinalis, its effects in gonorrhoea, —— I. 433.

Sarcocele, species of, in Senegal described, — III. 426. V. 32

Sarsaparilla, obs. relative to, —— —— VIII. 283

Savigny, J. description of an instrument for the fistula in ano, XI. 228

Saunders, Robert, on the diseases of Boutan and Thibet, ibid. 274

————, Dr. William, on the red Peruvian bark, — III. 248

————, his experim. with opium in Syphilis, IV. 420

———— account of a new extract of bark, XI. 67

Saw, proper for amputation, described, —— VII. 237

Scarpa, Anton. Anatomicæ Annotationes, — I. 211. IX. 419

Scarlatina anginosa, accounts of, — — I. 377, 379. X. 7

Scheele, C. W. on air and fire, —— —— — I. 312

————, chemical processes by, — — II. 53, 132

Schmucker, J. L. surg. essays, I. 231, 237, 320, 332. II. 10, 110, 168

Schoepff, J. D. of the effects of opium in Syphilis, III. 221

Schotte, Dr. J. D. case of Sarcocele, —— III. 426. V. 32

————, on the synochus atrabiliosa, —— V. 260

Sciatica, experiments in, —— —— I. 86

Scull, cases of fracture of, I. 324, 325. II. 13, 17, 18, 175, 176. IV. 72. V. 278. VII. 152. VIII. 411. X. 277. XI. 370

Scurvy, curious effects of, — — — — II. 117, 127

———— fact relative to the generation of, —— VII. 342

————, inquiry into the source of the symptoms of, III. 45

Senna, round leaved, described, —— — VIII. 236

Senses, external, curious facts relative to, —— III. 20

Sensibility,

Senfibility, morbid, inftances of, —— —— I. 177
Seton, ufe of, in hydrophthalmia, ——— —— *ibid.* 348
Sheep, method of bleeding them, defcribed, ——— III. 355
Sheldon, John, hiftory of the abforbent fyftem, —— V. 157
Shoes, differtation on the beft form of, ——— IV. 343
Sienna, Atti dell Academie di, —— ——— III. 43
Simmons, Dr. S. F. cafe of a caries of the fpine, — — I. 271
——————, obf. on gonorrhoea, —— —— II. 233
—————————the nat. hift. of the Peruvian bark, IV. 304
——————, account of the hydrocephalus which occurs in Mania, —— —— —— VI. 159
————————— an epidemic catarrh, — IX. 335
Skeete, Dr. Tho. cafe of effufion of bile, —— VI. 274
——————, experiments and obf. on Peruvian bark, VII. 254
——————, his reply to Dr. Irving, —— — VIII. 75
Skin, engravings to illuftrate the difeafes of, recommended, X. 366
Small-pox, facts and obfervations relative to, II. 145. VI. 206. VII. 7, 63, 66, 163, 180. X. 263, 353
————, inftances of its affecting, the fœtus in utero, II. 204. III. 121. VII. 165
————, complicated with meafles, inftance of, —— IV. 301
Snowden, Dr. S. cafe of retention of urine, — — VII. 10
Soaps, acid, obfervations relative to, III. 359. V. 352
Societies, Meteorological, eftablifhment of, I. 432
Soda phofphorata, account of, ——— IX. 393
Solvents, remarks on, ——— II. 222. IV. 382
Spalanzani, Abbé, his experiments on digeftion, III. 309
Sparrow, Richard, cafes by, —— —— IX. 109
Spence, G. on the difeafe confequent to tranfplanting teeth, X. 243
Spielman, J. R. Pharmacopeia generalis, — IV. 264
Spine, cafe of a curvature of, ——— VI. 358
Spirits, effects of an immoderate ufe of, ——— VII. 33
Spleen, cafe of enlargement of, ——— V. 186
Sternum, cafe of a fracture of, ——— VIII. 391
Stevenfon, James, cafe of a fatal fuppreffion of urine, IX. 382
————, Dr. William, cafes in medicine, —— III. 182
Stimuli, diftinctions of, ——— — I. 176
Stockholm, memoirs of the academy of, III. 20, 142
Stoellers, F. C. cafes medical and chirurgical, II. 36
Stomach, inftances of fatal affections of, IV. 43. IX. 385. XI. 17, 343
Strack, Carolus, de Crufta Lactea, ——— II. 187
———————— tuffi convulfiva, ——— *ibid.* 398
Strammonium, its medicinal effects, ——— II. 295
Sublimate, corrofive, external ufe of, recommended in Syphilis, IV. 265
——————, large quantity of it, fwallowed, VI. 271
Sudor Anglicus, fuppofed to refemble epidemic catarrh, IX. 373
Sue, M. Effais fur les accouchemens, ——— II. 351
Suette, of Picardy, what, ——— IV. 54
Sugar, acid of, remarks on, — — I. 81
———, obf. relative to, —— —— —— VIII. 281
Sulphur, fteams of, faid to produce dyfentery, II. 383

Surgery

Surgery, hiſtory of, ——— III. 260, 337

Sutures, of the cranium, ſuppoſed uſe of in the foetus, V. 362

Sweden, obſervations on the climate of, — III. 142

Swediar, Dr. F. caſe of a white ſwelling, — I. 194

——— cherry ſtone extracted from an abſceſs of the abdomen, — — — II. 337

——— hooping cough, — ibid. 408

——— a fungus of the bladder, III 194

———, on the venereal diſeaſe, — VI. 82

Swieten, Baron van, his poſthumous works, — IV. 145

Sympathy, treatiſe on, ——— — I. 284

Synochus, conſidered as a variety of Typhus, X. 125

Syphilis. See Lues.

### T.

Tabes chyluritica, caſe of, — — — II. 36

Tænia, ſyſtematic diviſion of the genus of, — VI. 78

———, remedies in caſes of, ——— II. 175. III. 157

———, hydatigena, deſcribed, — ——— X. 77

———, in the brain of ſheep, account of, IV. 111

Tamarind tree, deſcribed, — — — VIII. 287

Tanjore antidotes, for venomous bites, account of, X. 283

Tartar, cream of, its efficacy in dropſy, ——— I. 91

———, emetic, modes of preparing it, III. 358, 420

———, effects of a large doſe of, — IX. 61

Teeth, obſervations on their growth, III. 221, 264

———, tranſplanted, diſeaſe occaſioned by, VII. 205, 216. X. 243

Tendo Achillis, caſe of a rupture of, ——— VIII. 304

Tertian, caſe of, with uncommon ſymptoms, I. 395

Teſticles, their ſituation and deſcent in the foetus, ibid. 376

———, diſeaſed, cured by electricity, ——— VII. 247

Tetanos, obſervations relative to, VII. 424. X. 299

Thebaic tincture, its efficacy in ophthalmia, ——— I. 119

Thibet, account of the diſeaſes of, — — XI. 274

Thompſon, Sir Benj. expts. on ſubſtances uſed in cloathing, IX. 201

Thomſon, Dr. Alexander, on nervous diſorders, II. 347

Tibia, fiſſure of, — — — I. 329

———, inſtance of an application of the trepan to it, X. 80

Tickell, William, hiſtory of vitriolic æther, — VI. 337

Tin, chemical inquiries relative to, — — II. 359

Tipioca, accounts of, — — VIII. 263, 295. IX. 67

Tiſſot, M. on diſorders of the nerves, I. 32, 98, 174

Tobacco, account of its medicinal properties, VI. 185

Tomlinſon, T. on the cure of the hydrocele, — VIII. 119

Tooth, account of an excreſcence connected with one, II. 37

Torre, Padre della, his obſervations on the blood, III. 218

Toxicodendron, experiments on the poiſonous effects of, V. 20

Tranſmutation of metals, experiments relative to, III. 328

Trichiaſis, obſervations relative to, — I. 121. III. 5

Trochar, elaſtic, account of, ——— ——— I. 418

———, curved, for puncturing the bladder, deſcribed, XI. 356

Trye, C. B. account of an operation on the elbow joint, XI. 29
Tubercles, of the lungs, how formed, —— III. 387
Tufts, Hon. Cotton, on the horn diftemper in cattle, VII. 305
Tumour, encyfted, between the bladder and rectum, cafes of, VI. 325. XI. 13
——, obfervations on the treatment of, III. 10
——, flatulent, of the head, cafe of, —— VI. 424
——, fteatomatous, fingular one in the thorax, I. 387
Turner, T. cafe of amputation in the middle of the foot, IX. 54
—— a fuppreffion of urine, —— XI. 7
—— remarks on the paracentefis of the bladder, *ibid.* 14
Tuffis convulfiva, nature and treatment of, —— II. 398
——, productive of emphyfema, —— *ibid*, 408
Typhus nervofus, experiments in, —— —— I. 4

### V.

Vagina, cafe of gangrene of, —— —— I. 335
——, clofed by a thick membrane, —— —— III. 159
——, prolapfus of, hindering delivery, —— I. 335
——, inftance of its being deficient, —— —— II. 178
Vaux, George, his edition of Mr. Elfe's works, —— III. 380
Vegetables, poifonous. See poifons.
——, their effects in purifying the air, —— II. 2
Vein, coronary, its orifice defcribed, —— —— II. 35
——, ruptured, cafe of, —— —— —— VI. 141
Venæfection, obfervation on the abufe of, —— I. 179
——, numerous inftances of, in one patient, — X. 345
Venereal difeafe, See Lues.
Verbafcum, its efficacy in diarrhoea, —— —— I. 96
Vertebræ, diflocation of, —— —— I. 326, 327, 401
Vicq D'Azyr, M. anatomical obfervations by, II. 105. III. 276, 277, 279. V, 127, 340
Viola tricolor, recommended in the crufta lactea, II. 189
Viper, effects of its bite, —— —— —— III. 145
——, remarks on the poifon of, —— —— V. 3, 416
Virus, venereal, experiments with, —— —— III. 214
Vifcera, tranfpofed, cafe of, —— —— X. 178
Vifion, inftance of a peculiarity of, — —— VIII. 306
Vitriol, blue, efficacy of, in dropfy, — I. 266. X. 149
Ulcers, internal, cured by the ufe of lime water, — II. 16
—— of the legs, remarks on, —— —— IV. 254
——, putrid, obfervations on, —— —— VI. 373
Underwood, Dr. M. on ulcers of the legs, —— IV. 254
—— the difeafes of children, VI. 318
—— effects of crude mercury, VIII. 85
——, cafe of an extra-uterine fœtus, *ibid.* 321
Voice, organ of, its ftructure, —— —— V. 340
Vomica, cafes of, —— —— —— III. 11
——, remarks on the formation of, — — *ibid.* 387

Urethra, female, excrefcence in, — — — VII. 160

———, fiftulas of, not always venereal, — — III. 14

Urine, cafes of a fuppreffion of, VII. 10. IX. 382. XI. 7, 115, 118, 121, 349

———, fufible falt of, manner of preparing it, — V. 225

———, experiments on, in diabetes, I. 89. IX. 290. XI. 225

———, incontinence of, cafe of, ——— ——— VI. 417

———————, propofed remedy for, ——— I. 135

Uterus, double, inftances of, ——— ——— III. 19, 425

———, imperforated, cafe of, ——— ——— IV. 243

———, inverted, cafes of, ——— ——— II. 112. VI. 367

———, retroverted, faid to be known to the ancients, III. 348

———————, cafes of, ——— — I. 392. XI. 380

———, rupture of, facts and obfervations relative to, V. 424. VI. 92. VIII. 356

———, account of a fingular ftricture of, — — I. 384

——————— fubject in which it was deficient, II. 178

Uva Urfi defcribed, ——— ——— ——— IV. 264

W.

Wall, Dr. Martin, on felect fubjects of chemiftry and phyfic, V. 105
Walnuts, efficacy of the green outer fhell of, in ulcers, X. 319
Walter, J. G. de Sectione Synchondrofeos Pubis, — III. 366
Ware, James, on the ophthalmy, ——— ——— I. 115
Warren, Dr. John, on the ufe of the fox glove in dropfy, VI 145
——————— hydrocephalus internus, IX. 122
———, John, cafe of a tumour containing hair, ——— VII. 298
Water, of the Bath, in Jamaica, account of, ——— II. 413
———, cold, obfervations on its medicinal ufes, II. 18. IV. 350, VI. 264. VII 108, 109, 123. VIII. 173
———, decompofition of, — — — IV. 414. V. 202
———, fea, obfervations relative to, ——— ——— I. 75
Waters, mineral, works relative to, I. 74, 294, 369. II. 68, 139, 320. III 110, 111, 134, 284, 327, 360, 361. IV. 30, 218. V. 218, 336. VI. 331. VII. 100, 102, 223. VIII. 427. IX. 101, 214, 326, 416, 427. X. 218, 220, 427. XI. 213.
———————, methods of analyzing, ——— ——— II. 321
——————— imitating, ——— ——— I. 75
Wathen, Jonathan, of the fiftula lachrymalis, — — II. 245
Watfon, Henry, on the paracentefis of the bladder, XI. 349
———, Dr. Richard, chemical effays, ——— ——— II. 422
——— William, on the effects of tranfplanted teeth, VII. 216
Watt, James, on the conftituent parts of water, — VI. 175
Webfter, Dr. Charles, his life of Dr. Archibald Pitcairne, V. 326
———, J. S. cafe of a peculiarity of vifion, — VIII. 306
Wedgewood, Jofiah, his meafure of the higher degrees of heat, IV. 225
Weed, a fpecies of fever fo called, what, — — I. 168
Welchman, J. cafe of a fection of the fymphyfis pubis, XI. 46
Wens, cafe of a man covered with, ——— ——— VI. 33

Werner,

Werner, P. on the practice of inoculation in Algiers, XI. 141

——, miscellaneous observations, —— ibid. 143

——, case of diabetes, —— —— ibid. 221

Wheat, malted, meal of, recommended instead of sweetwort, V. 325

White, Charles, his account of Captain M——'s case, IV. 159

——, on a swelling of the lower limbs in lying-in women, —— —— —— — V. 50

—— gangrenes, — — — — XI. 167

——, Dr. Robert, case of hydrocephalus, —— III. 402

——, on the treatment of cancers, — V. 70

——, Dr. William, on the bills of mortality at York, IV. 2

Whitehurst, John, case of palsy cured by electricity, V. 77

Wichmann, J. J. of the insect found in the itch, —— IX. 28

Wier, Edward, remarks on hydrocephalus internus, IV. 78, 393

Wigglesworth, E. on American bills of mortality, VII. 316

Wilkinson, George, case of fistula in perinæo, —— IX. 378

—— hepatitis, — — — X. 142

—— fractured ribs, — — XI. 130

—— scull, —— ibid. 376

——, observations on the Angustura bark, ibid. 331

Willan, Dr. Robert, on the Croft water, —— III. 327

——, letter to, concerning the byssus lanuginosa, III. 420

——, account of a new species of byssus, IV. 421

——, case of obstruction of the bowels, V. 401

—— chorea Sancti Viti, —— VII. 187

—— singular termination of dropsy, ibid. 189

——, of the use of arsenic in intermittents, VIII. 191

—— effects of the Angustura bark, XI. 44

Williams, Dr. A. account of a new species of bark, X. 158

Wilmer, B. on vegetable poisons, — — — I. 336

——, his manner of treating wounds of the head, V. 440

——, case of hydatids discharged from the uterus, VIII. 382

Wilson, Patrick, his experiments on cold, — —— I. 433

Wintringham, Clifton, de morbis quibusdam, — III. 177

Withering, Dr. William, on the fox glove, —— VI. 298

Wltczeck, Ign. Casus peculiaris historia, — — VII. 332

Worms, in the meatus auditorius, cases of, — — III. 9

——, intestinal, treatise on, —— —— VI. 76

——, voided through wounds communicating with the intestines, VII. 251, 372

Wounds, gun-shot, cases of, I. 323, 325, 328. II. 112, 113, 172, 175, 177. VII. 138

——, observations on the treatment of, XI. 363

Wright, Dr. William, of the efficacy of blue vitriol in dropsy, I. 266. X. 149

—— use of cold bathing in the locked jaw, VI. 415

——, facts relative to the small pox, VII. 63

——, on cold bathing in fevers, — ibid. 109

—— the effects of vegetable acid and marine salt, —— —— —— —— VIII. 97

Wright,

Wright, Dr. William, on the medicinal plants of Jamaica, VIII. 217

Wriſberg, H. A. anatomical diſſertations by, I. 375, 376. III. 26

Y.

Yeaſt, artificial, method of preparing it, — — II. 139

Z.

Zimmermann, Dr. J. G. on experience in phyſic, — IV. 9

Zinc, chemical remarks on, — — — — II. 105

——, flowers of, their efficacy in epilepſy, inſtanced, VII. 52

END OF THE WORK.

